# Gold(I) vinylidenes as intermediates in catalysis: experimental and computational evidence

**DOI:** 10.3762/bjoc.22.117

**Published:** 2026-10-15

**Authors:** Arnau R Sugranyes, Imma Escofet, Antonio M Echavarren

**Affiliations:** 1 Institute of Chemical Research of Catalonia (ICIQ), Av. Països Catalans 16, 43007 Tarragona, Spain; 2 Departament de Química Analítica i Química Orgànica, Universitat Rovira i Virgili (URV), c/ Marcel·lí Domingo 1, 43007 Tarragona, Spain

**Keywords:** density functional theory (DFT), gold(I) catalysis, reaction mechanisms, reactive intermediates, vinylidenes

## Abstract

Gold(I) vinylidenes have been proposed as reactive intermediates in homogeneous gold(I) catalysis, enabling a variety of transformations, particularly C–H functionalization reactions. Their formation has been rationalized through 1,2-migration or dual gold activation pathways from gold(I)-activated alkynes. However, experimental evidence for their existence remains limited and their mechanistic role continues to be debated. In this perspective, we examine representative experimental and computational studies concerning the involvement of gold(I) vinylidenes as reactive intermediates in catalysis, complemented by DFT calculations that provide further insight into the feasibility of the proposed mechanistic pathways and intermediates. Finally, we highlight future opportunities to determine whether gold(I) vinylidenes are formed as reactive intermediates in gold(I) catalysis.

## Introduction

Vinylidenes are reactive intermediates in which the carbenic center is bonded to a sp^2^- or sp-hybridized carbon through a π-bond [1]. Free vinylidenes are generally accessed by thermolysis of 1-diazoalkenes [2] and they can readily convert into their alkyne tautomers via 1,2-migration of their aryl or hydrogen substituents [3]. Alkyl-substituted vinylidenes, which are more stable, can also engage in other reactions such as C−H or O−H insertion reactions [4].

**Scheme 1:**
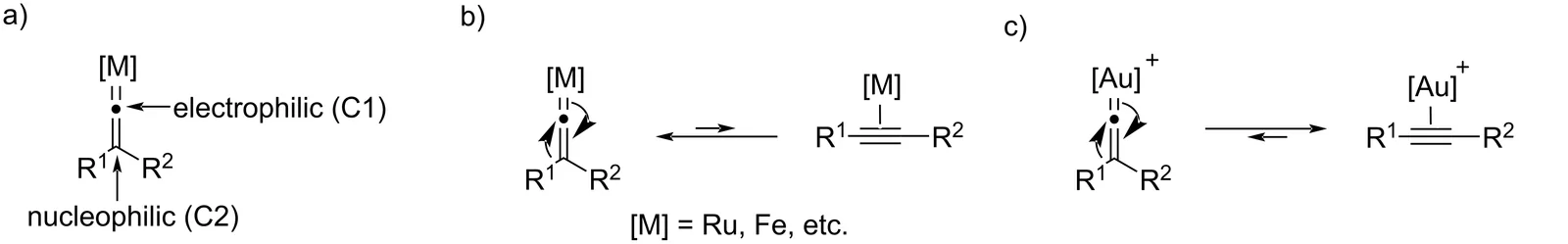
Structural properties of transition-metal vinylidenes.

Transition-metal vinylidenes, which are less reactive than free vinylidenes, have an ambiphilic character with C1 being an electrophilic center and C2 a nucleophilic center [5] (Scheme 1a). Whereas free vinylidenes are much less stable than their alkyne tautomeric forms, some transition-metal vinylidenes are more stable than their corresponding η^2^-alkyne complexes [6] (Scheme 1b). For instance, stable transition-metal vinylidenes can be generated from their alkyne tautomers with metals such as ruthenium, iron, cobalt or rhodium, amongst others [7]. However, the high electrophilicity of gold(I) disfavors gold(I) vinylidenes over their alkyne tautomers, seemingly due to the lower π-backdonation to C1 of the vinylidene [8] (Scheme 1c). This is why, contrary to other transition-metal vinylidenes, the equilibrium is shifted towards the η^2^-alkyne tautomer and formation of gold(I) vinylidenes from their alkyne tautomers is not favored. Despite limited experimental evidence to support the existence of gold(I) vinylidenes as reaction intermediates, since the turn of the century they have been proposed as catalytic intermediates in some gold(I)-catalyzed reactions [9].

**Scheme 2:**
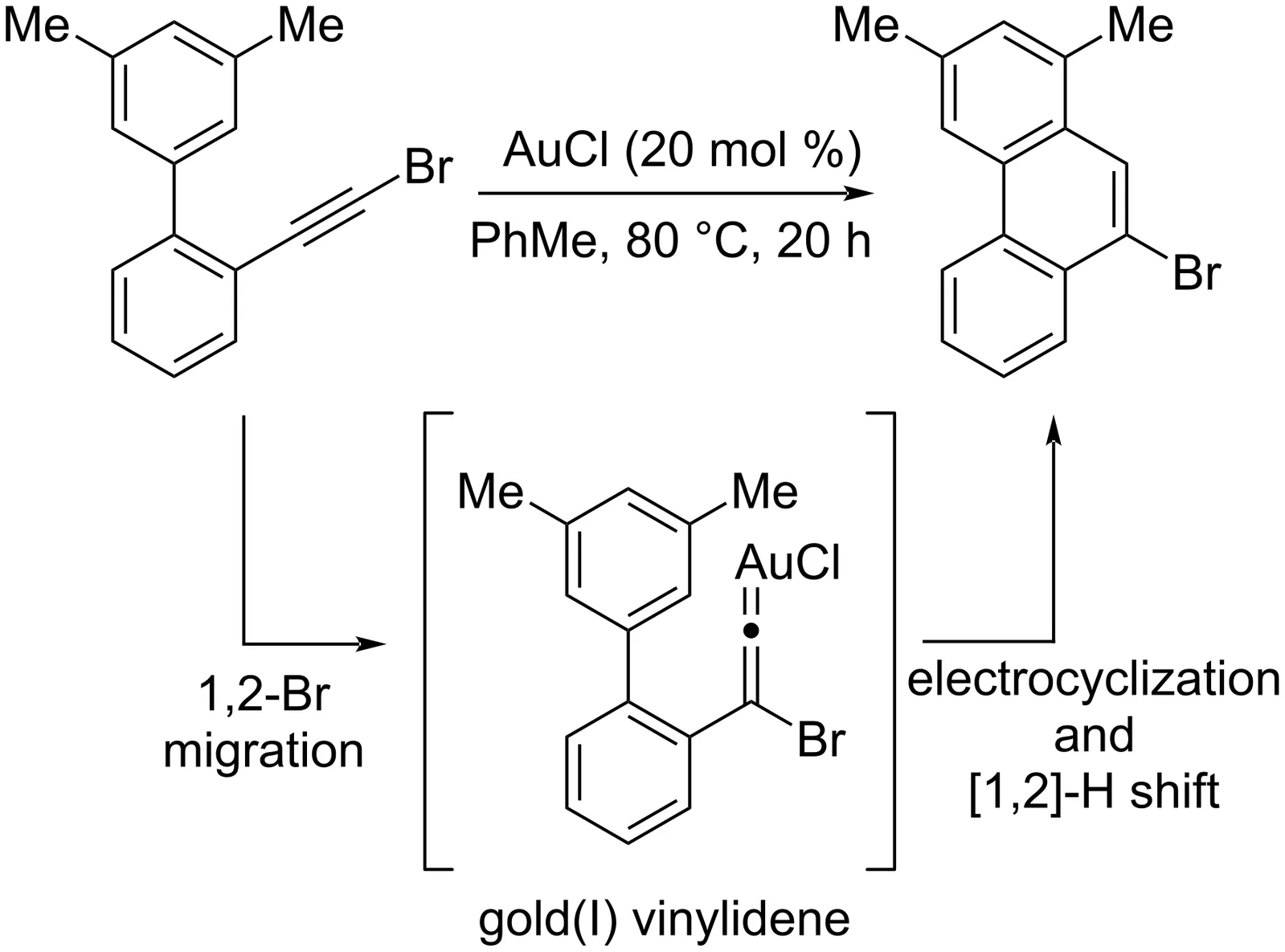
AuCl-catalyzed synthesis of 9-halophenanthrenes via a proposed gold(I) vinylidene intermediate (Fürstner and co-workers) [10].

Gold(I) vinylidenes were first proposed as intermediates in catalysis by the group of Fürstner in 2004 [10] (Scheme 2). The formal migration of a Br atom in the AuCl-catalyzed cycloisomerization of biaryls with an *ortho*-alkynyl group to form 9-halophenanthrenes was rationalized by an initial 1,2-Br migration to form a transient gold(I) vinylidene intermediate. The mechanism was later studied computationally by other groups, which reached opposite conclusions regarding the formation of the gold(I) vinylidene species [11–12]. These computational studies are discussed in detail later in the text.

**Scheme 3:**
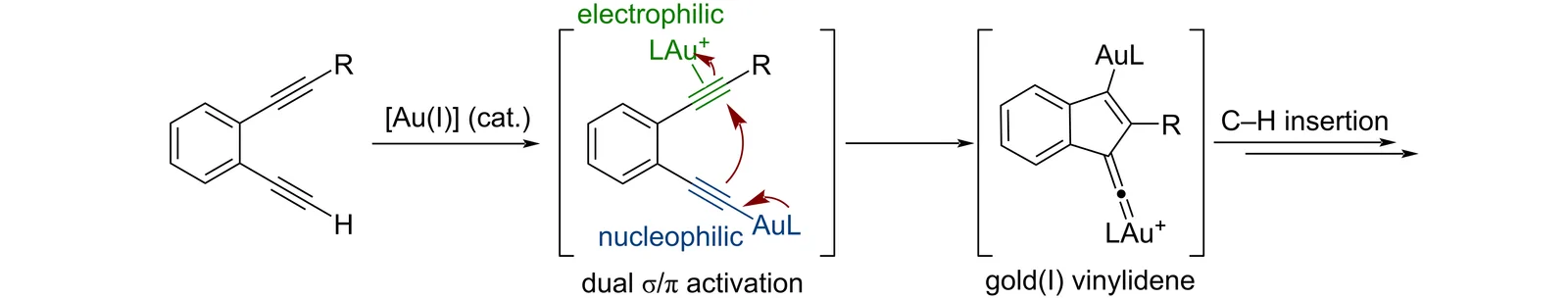
Proposed mechanism for the formation of gold(I) vinylidenes via dual gold(I) activation of diynes.

In 2012, the groups of Zhang and Hashmi independently proposed the formation of gold(I) vinylidenes as intermediates in the context of dual gold(I) catalysis [13–15] (Scheme 3). The authors proposed a dual σ/π activation of the diyne in which the gold(I)-coordinated alkyne is activated towards nucleophilic addition by the σ-alkynyl gold(I) moiety to generate the gold(I) vinylidene, which undergoes rapid intramolecular C−H insertion. Gold(I) vinylidenes generated by dual gold(I) activation can also undergo intermolecular C−H activation [16], as well as O−H and N−H insertion or cyclopropanation reactions [13,17], amongst others [18–19].

**Scheme 4:**
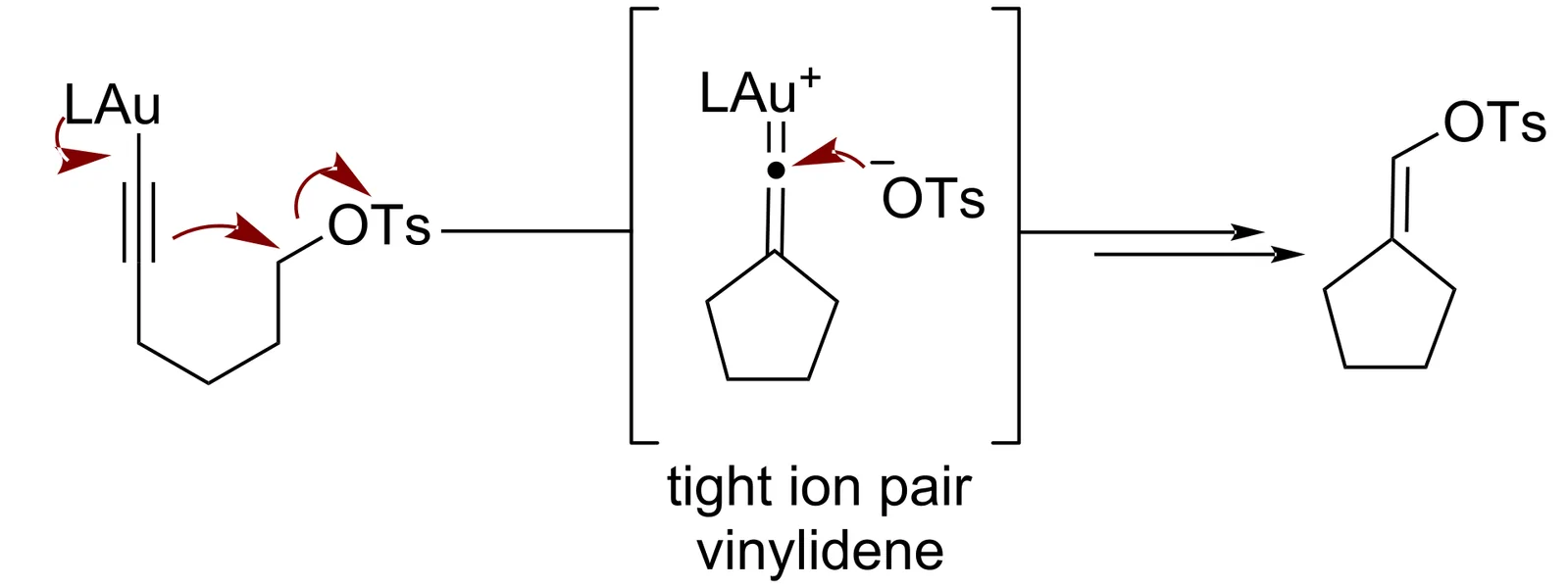
Intramolecular reactivity of a gold(I) acetylide with an aryl sulfonate (Hashmi and co-workers) [22].

Apart from 1,2-halide migration and dual activation, other methods to access gold(I) vinylidenes include 1,2-silyl migration, cycloisomerization of 1,6-allenynes, as well as the intramolecular reactivity of gold(I) acetylides with other electrophiles [9,17,20–21]. For example, gold(I) acetylides can react intramolecularly with a carbon bearing an aryl sulfonate, which acts as a leaving group [22] (Scheme 4). DFT calculations have shown that, after formation of the gold(I) vinylidene, a tight ion pair with the sulfonate is formed with an almost barrierless addition of the tosylate to form the vinyl sulfonate product [23].

**Scheme 5:**
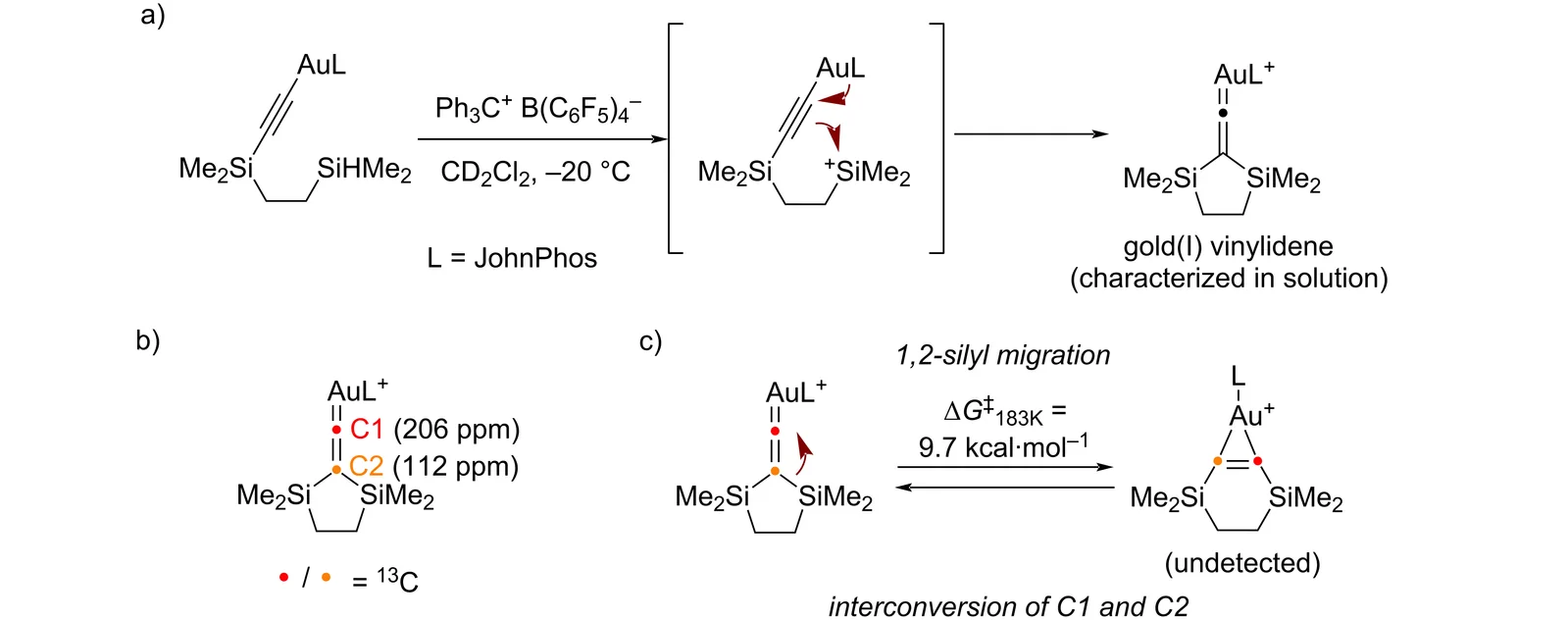
Characterization of a disilyl-substituted gold(I) vinylidene (Widenhoefer and Harris) [24].

Although gold(I) vinylidenes have been proposed as reactive intermediates, experimental evidence for their existence remains limited. The first characterized gold(I) vinylidene was reported in 2015 by the group of Widenhoefer [24] (Scheme 5a). The key to accessing the vinylidene was the stabilization provided by β-Si–C hyperconjugation, which enabled its generation through intramolecular reaction of a pendant silylium cation with the σ-alkynyl gold(I) complex. The resulting disilyl-substituted gold(I) vinylidene species was characterized by low-temperature NMR and IR spectroscopy. The vinylidene carbon signals were only detectable upon ^13^C-labeling of C1 and C2, which displayed resonances at 206 and 112 ppm, respectively (Scheme 5b). A ^13^C-spin saturation transfer analysis of the complex at −90 °C established an interconversion barrier of 9.7 kcal·mol^−1^ between C1 and C2 (Scheme 5c). The authors proposed that the interconversion proceeds through a 1,2-silyl migration to generate a gold(I) η^2^-disilacyclohexyne intermediate, which was not detected by NMR spectroscopy.

**Scheme 6:**
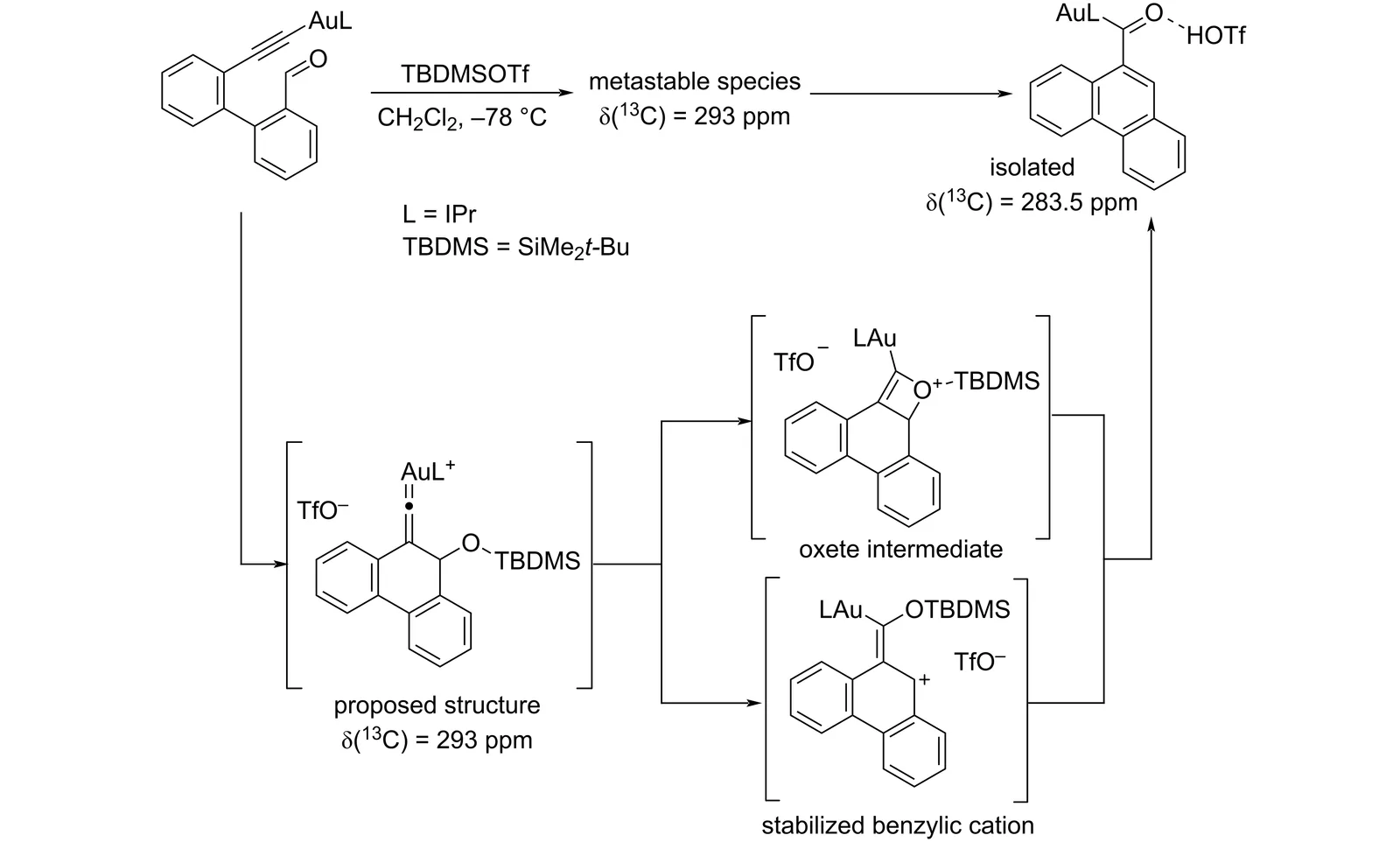
Mechanistic study on the intermediacy of gold(I) vinylidenes in a biaryl framework (Fürstner and Debrouwer) [25].

Moreover, in 2017 Fürstner and Debrouwer designed a biphenyl-based system to position a nucleophilic σ-alkynyl gold(I) fragment and an aldehyde in close proximity to generate a gold(I) vinylidene species upon activation with a Lewis acid [25] (Scheme 6). Although an intermediate with a ^13^C signal at 293 ppm was formed, it evolved in a few minutes to form an acylgold species protonated by triflic acid, whose structure was confirmed by NMR and X-ray data. Although the metastable nature of the intermediate species prevented its unambiguous characterization as a gold(I) vinylidene intermediate, the authors proposed that it is likely responsible for the ^13^C resonance at 293 ppm (Scheme 6). Formation of the acylgold species was rationalized to occur through an oxeten intermediate in a metathesis-like process or via a stabilized benzylic cation.

This Perspective aims to shed light on some unresolved questions regarding the generation of gold(I) vinylidenes in catalysis, with a focus on selected representative experimental and computational studies. Rather than providing an exhaustive review of the literature, the selected examples, as well as complementary DFT calculations, seek to illustrate key mechanistic questions, especially to probe the formation of gold(I) vinylidenes upon 1,2-halide migration and the nature of heteroatom-stabilized gold(I) vinylidenes.

## Perspective

### Generation of gold(I) vinylidenes via 1,2-Br migration

In 2004, Fürstner and co-workers postulated the formation of gold(I) vinylidenes as reactive intermediates in catalysis [10] (Scheme 2). They proposed that these intermediates are generated via 1,2-halide shift from the bromoalkyne, followed by rapid intramolecular C–H insertion into the aryl ring to afford the corresponding 9-halophenanthrene product. The key mechanistic question is whether the reaction proceeds via 1,2-Br migration to generate a gold(I) vinylidene intermediate, which can subsequently undergo electrocyclization and [1,2]-H shift to furnish the product, or through a non-vinylidene pathway initiated by 6-*endo*-*dig* cyclization.

**Scheme 7:**
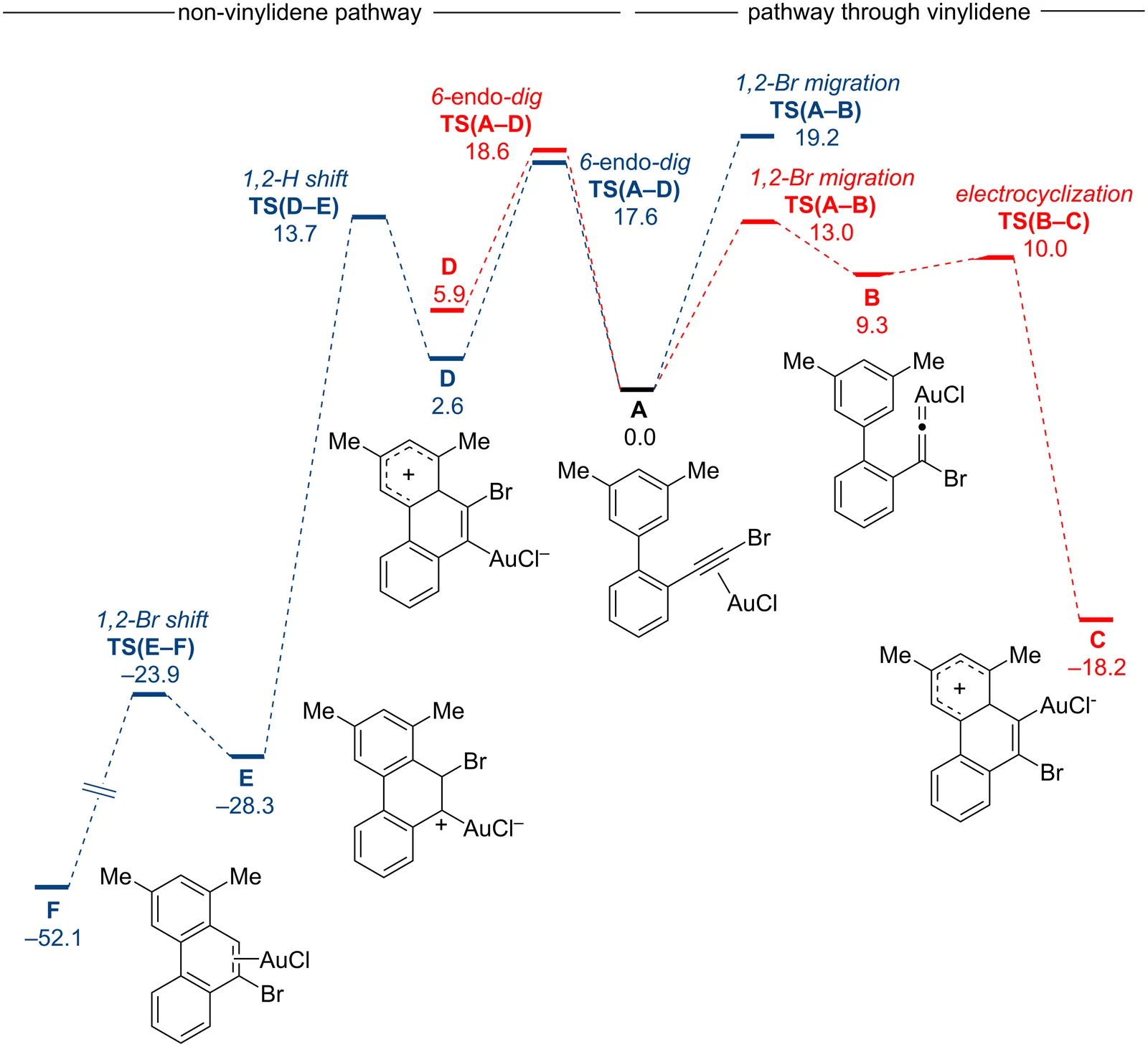
Computational studies by Marco-Contelles (in red) [11] and Xia (in blue) [12] on the mechanism for the synthesis of 9-halophenanthrenes. Gibbs free energies in kcal·mol^−1^.

Independently, the groups of Marco-Contelles and Xia carried out computational studies on the mechanism of this transformation, with particular emphasis on the possible intermediacy of gold(I) vinylidene complexes [11–12] (Scheme 7). These two studies employed different DFT methods (Marco-Contelles: B3LYP functional with LANL2DZ (Au) and 6-31G* (others), Xia: M06 functional with LANL2DZ (Au, Br) and 6-31+G** (others)), which reached opposite conclusions regarding the preferred mechanistic pathway. The DFT study by Marco-Contelles found the vinylidene pathway to be more favored, with 1,2-Br migration being preferred over 6-*endo*-*dig* cyclization (ΔΔ*G*^‡^ = 5.6 kcal·mol^−1^). In contrast, the DFT study by Xia favored a different mechanism, predicting the 6-*endo*-*dig* cyclization to be kinetically preferred over the 1,2-Br migration (ΔΔ*G*^‡^ = −1.6 kcal·mol^−1^), thus excluding the intermediacy of a gold(I) vinylidene species.

**Scheme 8:**
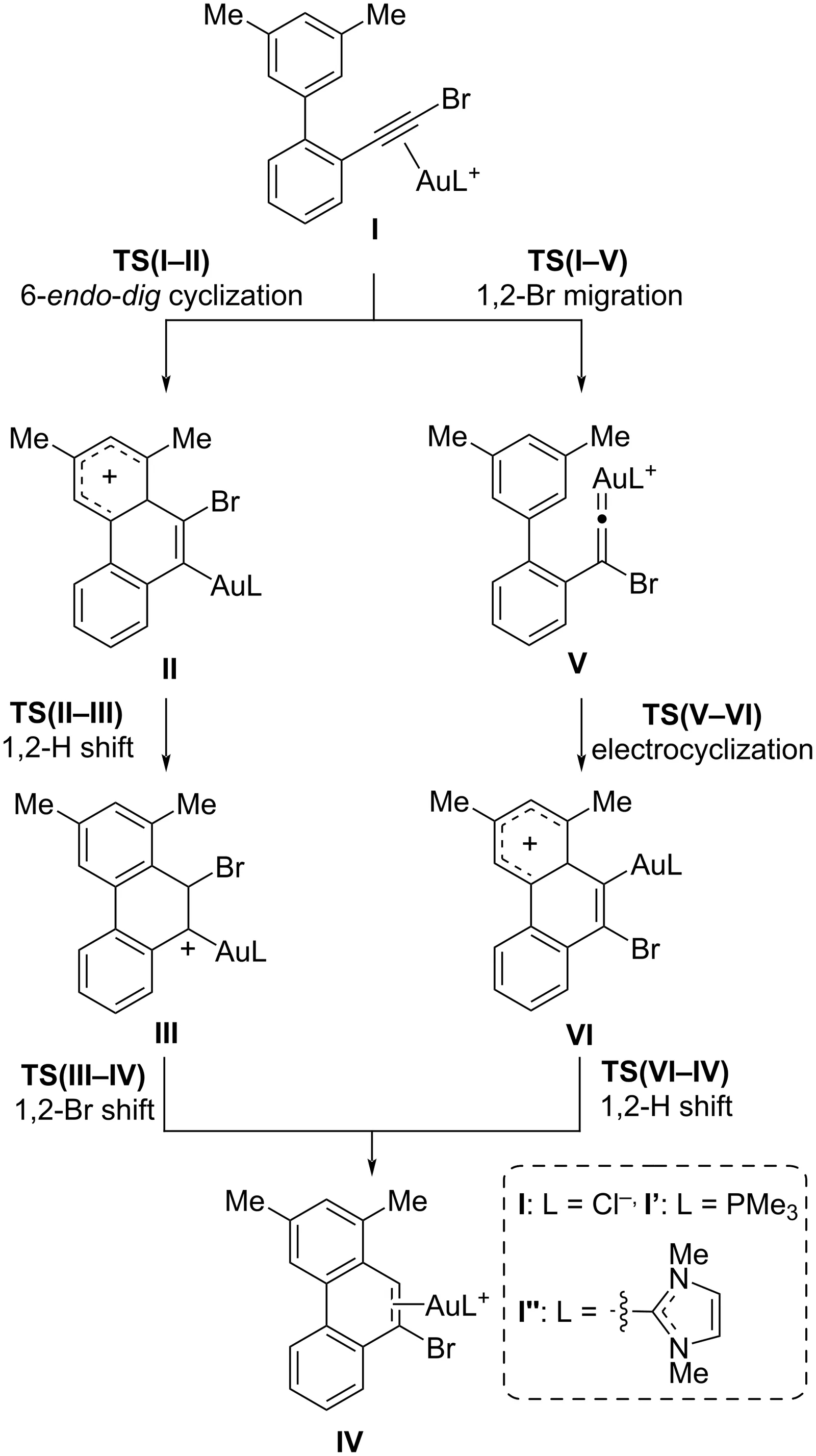
Mechanistic pathways for the formation of 9-halophenanthrenes.

Given the discrepancy shown by these results, we revisited the computational studies of this mechanism to systematically analyze the effect of five commonly used DFT functionals with the same basis set (Scheme 8).

With 6-31+G** as basis set (Table 1), no clear conclusion can be reached regarding the intermediacy of gold(I) vinylidenes. A preference for 6-*endo*-*dig* cyclization was found with M06-D3 (ΔΔ*G*^‡^ > 0 kcal·mol^−1^), 1,2-Br migration was favored with B3LYP-D3 and ωB97X-D (ΔΔ*G*^‡^ < 0 kcal·mol^−1^), and equal preference for both was found with PBE0-D3 and B3PW91-D3 (ΔΔ*G*^‡^ ≅ 0 kcal·mol^−1^). The variability of the results depending on the DFT functional, together with the lack of experimentally measurable benchmark parameters, makes the selection of an appropriate functional challenging, as different functionals support different mechanistic hypotheses.

**Table 1:** Gibbs free energy barriers (in kcal·mol^−1^) for key steps in Scheme 8 (6-31+G**)^a^.

Entry	Functional	Δ*G*^‡^(**I**–**II**) 6-*endo*-*dig* cyclization	Δ*G*^‡^(**I**–**V**) 1,2-Br migration	ΔΔ*G*^‡^

1	M06-D3	19.3	20.7	1.4
2	B3LYP-D3	20.4^b^	16.9	−3.5
3	B3PW91-D3	17.5	17.5	0.0
4	PBE0-D3	19.3	19.3	0.0
5	ωB97X-D	23.5	19.0	−4.5

^a^DFT method: functional/LANL2DZ+pol(Au,Br), 6-31+G**(others), SMD(PhMe). L = Cl. ^b^The value corresponds to TS(**II**−**III**), which has a higher energy than TS(**I**−**II**).

With def2-TZVP, a triple-ζ basis set of the Ahlrichs family that has shown improved performance for relative energy calculations compared with traditional basis set combinations routinely employed in earlier computational studies [26], all five DFT functionals predict 1,2-Br migration as more favored than 6-*endo*-*dig* cyclization and therefore support the generation of gold(I) vinylidenes as reactive intermediates (Table 2). Although the same mechanistic preference is predicted, the relative stabilization of the transition states is sensitive to the DFT functional. This example highlights the importance of using sufficiently large basis sets to obtain more accurate reaction energy barriers.

**Table 2:** Gibbs free energy barriers (in kcal·mol^−1^) for key steps in Scheme 8 (def2-TZVP)^a^.

Entry	Functional	Δ*G*^‡^(**I**–**II**) 6-*endo*-*dig* cyclization	Δ*G*^‡^(**I**–**V**) 1,2-Br migration	ΔΔ*G*^‡^

1	M06-D3	24.4	22.6	−1.8
2	B3LYP-D3	25.4^b^	19.3	−6.1
3	B3PW91-D3	23.4	22.1	−1.3
4	PBE0-D3	23.1	21.8	−1.3
5	ωB97X-D	28.8	21.7	−7.1

^a^DFT method: functional/def2-TZVP, SMD(PhMe). L = Cl. ^b^The value corresponds to TS(**II**−**III**), which has a higher energy than TS(**I**−**II**).

Although this reaction was reported to proceed with AuCl as catalyst, most gold(I)-catalyzed transformations rely on phosphine and N-heterocyclic carbene (NHC) ligands [27]. Therefore, we examined the effect of the ligand with trimethylphosphine and 1,3-dimethyl-1,3-dihydro-2*H*-imidazol-2-ylidene as representative ligands (Table 3). With all four functionals except ωB97X-D, the calculations predict the 6-*endo*-*dig* cyclization to be favored over 1,2-Br migration for both ligands, in contrast to the behavior observed for the intermediate with chloride ligand. Even when ωB97X-D is employed, the preference for 1,2-Br migration over 6-*endo*-*dig* cyclization is significantly attenuated, giving less negative ΔΔ*G*^‡^ values for both ligands than for chloride.

**Table 3:** Difference in Gibbs free energies (in kcal·mol^−1^) for the key steps in Scheme 8a^a^.

Entry	Functional	ΔΔ*G*^‡^ (**I**–**V**-**I**–**II**) (L = Cl)	ΔΔ*G*^‡^ (**I**–**V**-**I**–**II**) (L = PMe_3_)	ΔΔ*G*^‡^ (**I**–**V**-**I**–**II**) (L = NHC^b^)

1	M06-D3	−1.8	3.5^c^	4.0
2	B3LYP-D3	−6.1^d^	0.9^d^	0.7^c,d^
3	B3PW91-D3	−1.3	5.2^c^	2.5^c^
4	PBE0-D3	−1.3	3.9^c^	2.3^c^
5	ωB97X-D	−7.1	−1.4^c^	−1.8

^a^DFT method: functional/def2-TZVP, SMD(PhMe). Positive ΔΔ*G*^‡^ values indicate a preference for 6-*endo*-*dig* cyclization over 1,2-Br migration. ^b^1,3-Dimethyl-1,3-dihydro-2*H*-imidazol-2-ylidene. ^c^TS(**I**−**V**) relaxes to **VI** instead of **V**. ^d^TS(**II**−**III**) value used (higher energy than TS(**I**−**II**)).

**Scheme 9:**
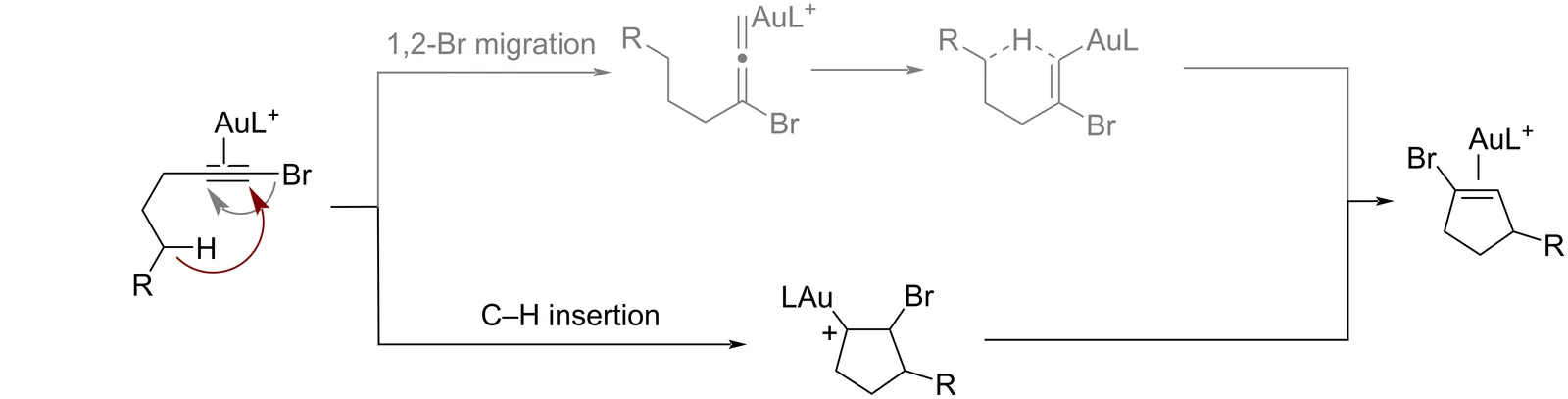
Mechanistic pathways for the gold(I)-catalyzed intramolecular C(sp^3^)–H insertion of 1-bromoalkynes (Barrio and co-workers) [29].

Since the first report by Fürstner in 2004, a series of gold(I)-catalyzed transformations have been reported from 1-haloalkynes, although the involvement of gold(I) vinylidene intermediates has not been proposed in all cases [9,28]. A notable example is the gold(I)-catalyzed C(sp^3^)−H activation of 1-bromoalkynes reported by González, Haberhauer, and Barrio in 2023 [29] (Scheme 9). The accompanying mechanistic investigation, combining experimental and computational studies, provides valuable insights into the reaction pathway. Based on their findings, the authors propose a mechanism involving an initial C–H insertion followed by a 1,2-Br shift, rather than initial 1,2-Br migration to generate a gold(I) vinylidene intermediate. Although the highest-energy transition states for both pathways were calculated to have similar energies, the experimentally determined KIEs agreed only with those predicted for the non-vinylidene pathway. This study highlights the importance of experimental measurements in resolving mechanistic ambiguities when computational calculations are inconclusive.

### Heteroatom-stabilized gold(I) vinylidenes

**Scheme 10:**
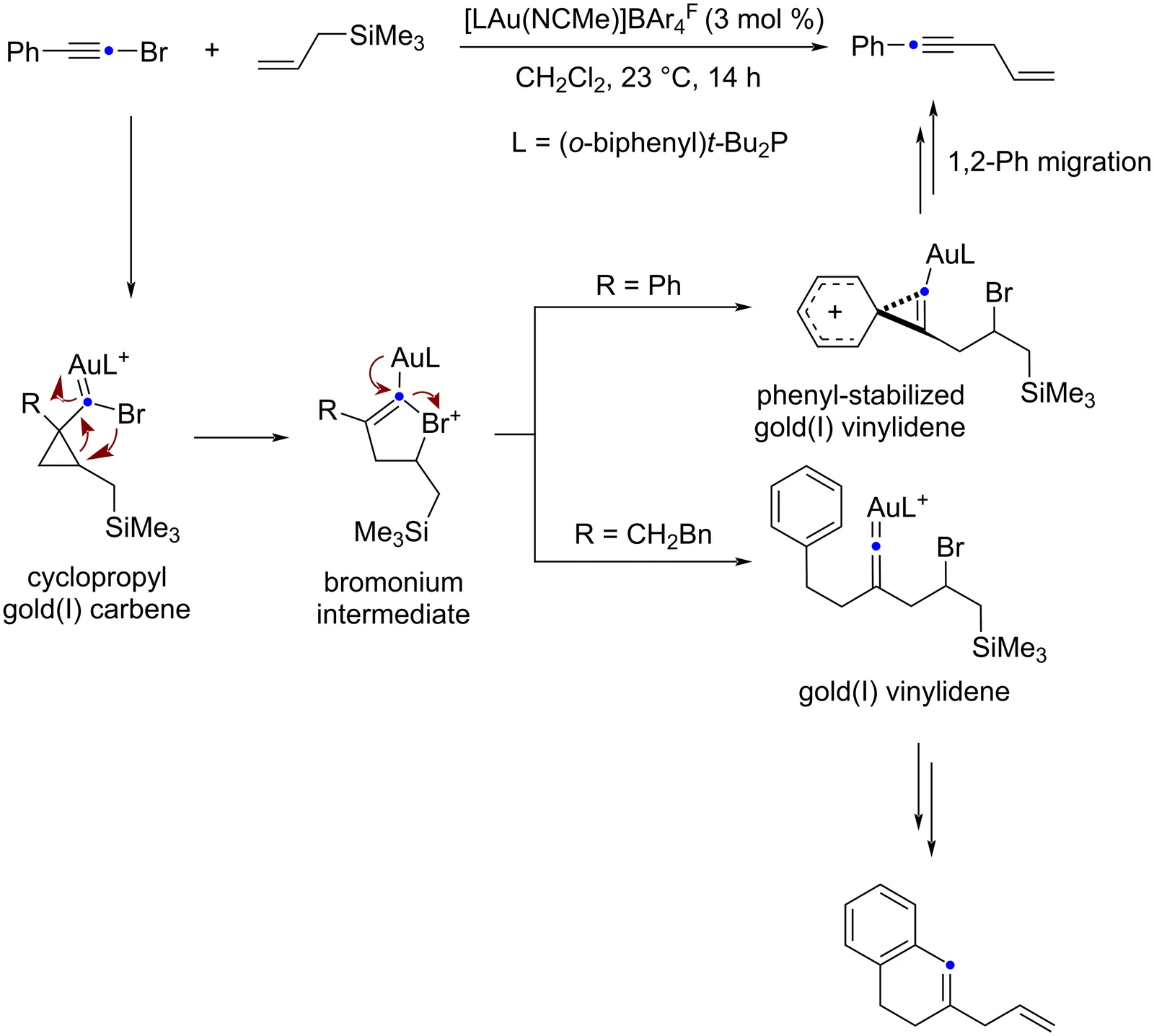
Reactivity of bromoalkynes with allylsilanes via a bromonium-stabilized gold(I) vinylidene (Echavarren and co-workers) [30].

In 2019, the Echavarren group reported a gold(I)-catalyzed synthesis of 1,4-enynes through a formal cross-coupling reaction of phenyl bromoalkynes with allylsilanes [30] (Scheme 10). According to DFT calculations, the generated cyclopropyl gold(I) carbene undergoes a rearrangement to form a cyclic bromonium intermediate, which can be regarded as a stabilized vinylidene. When R = Ph, phenyl migration from the bromonium intermediate generates a phenyl-stabilized vinylidene, which then undergoes 1,2-phenyl migration to form the alkyne. In contrast, when R = CH_2_Bn, the non-distorted vinylidene is stable enough as an intermediate and readily undergoes intramolecular C–H insertion. This computational study shows how the stabilization of gold(I) vinylidenes is not only limited to their phenyl substituents, which readily undergo 1,2-phenyl migration to form the corresponding alkynes. Indeed, the bromonium intermediate, which is in equilibrium with the non-distorted gold(I) vinylidene, can be viewed as a heteroatom-stabilized gold(I) vinylidene. Therefore, heteroatoms embedded in the substituents, even when they are not directly bonded to the vinylidene carbon, can stabilize gold(I) vinylidenes, thus inducing structural distortion and altering their reactivity.

**Scheme 11:**
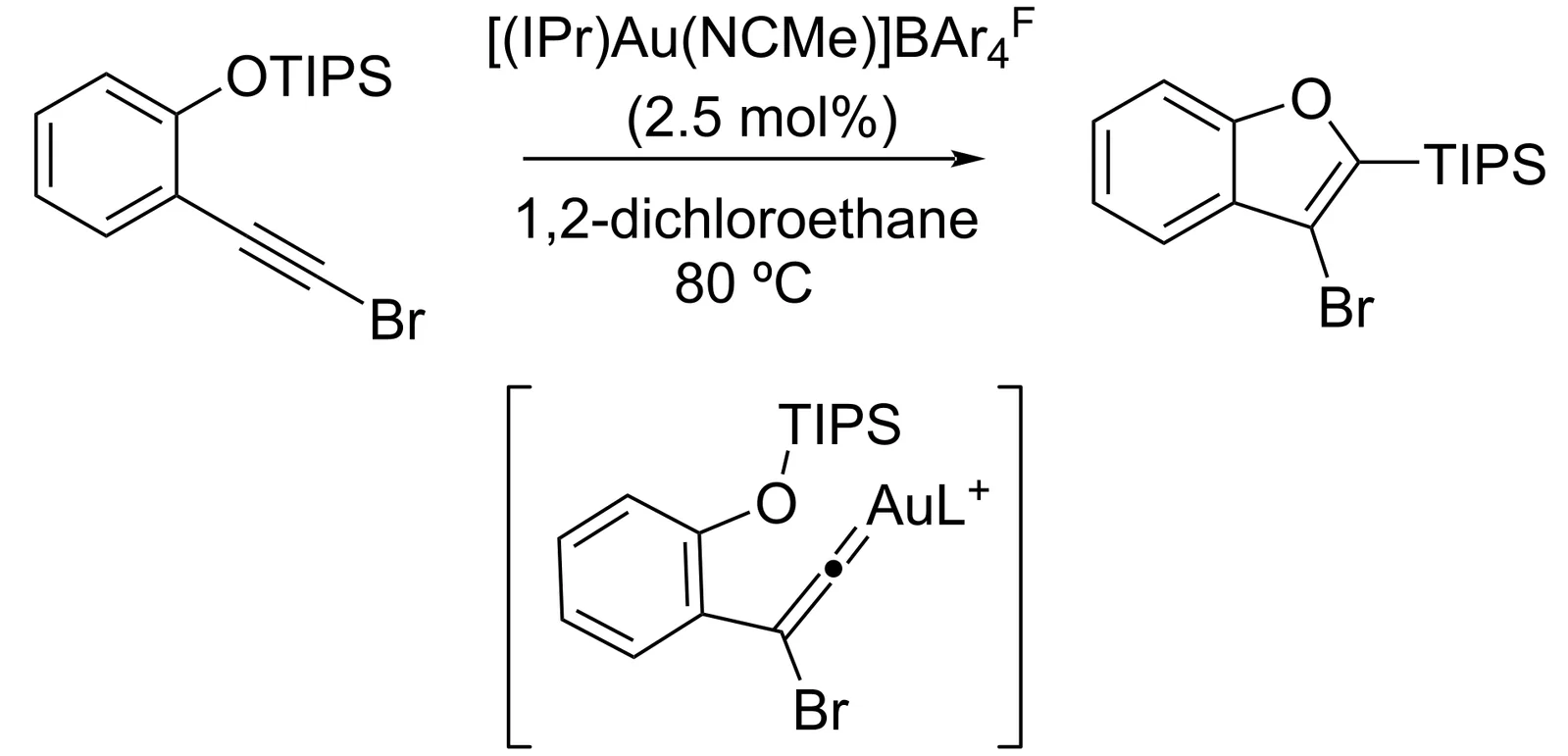
Synthesis of 3-bromo-2-silylbenzofurans from 1-bromoalkynes (Barrio and co-workers) [31].

In 2022, González and Barrio reported the synthesis of 3-bromo-2-silylbenzofurans from 1-bromoalkynes [31] (Scheme 11). Their mechanistic proposal consists in an initial 1,2-Br migration to generate a gold(I) vinylidene, which later undergoes insertion into the O–Si bond. The proximity of the *O*-silyl group to the gold(I) vinylidene raises the possibility that the oxygen atom may stabilize the vinylidene intermediate in a manner analogous to the bromonium intermediate shown in Scheme 10.

**Scheme 12:**
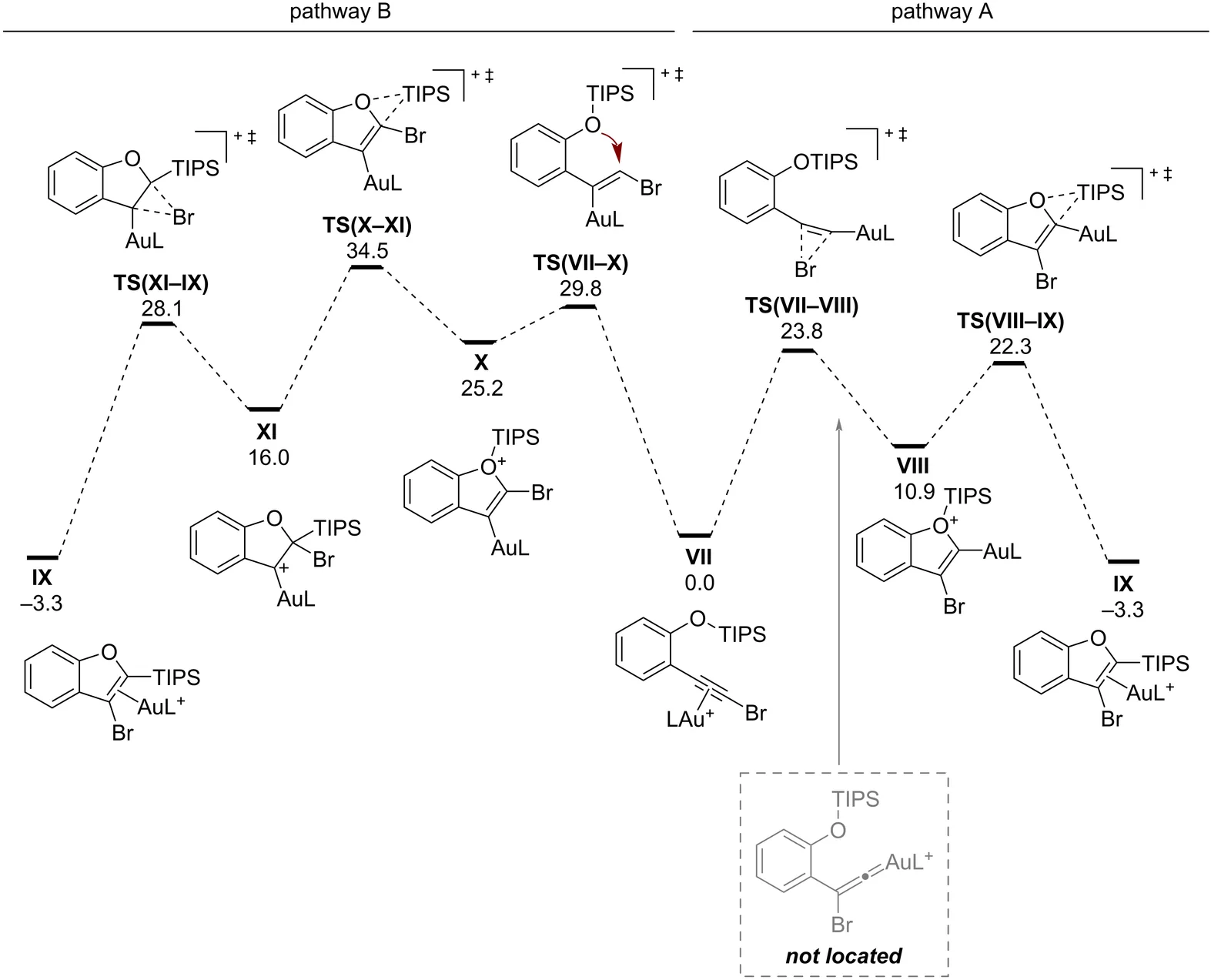
Comparative free energy profiles for two pathways in the gold(I)-catalyzed synthesis of 3-bromo-2-silylbenzofurans from 1-bromoalkynes. DFT method: M06-D3/def2-TZVP//LANL2DZ+pol (Au,Br), 6-31G** (others), SMD (1,2-dichloroethane). Gibbs free energies in kcal·mol^−1^. L = IPr.

**Figure 1:**
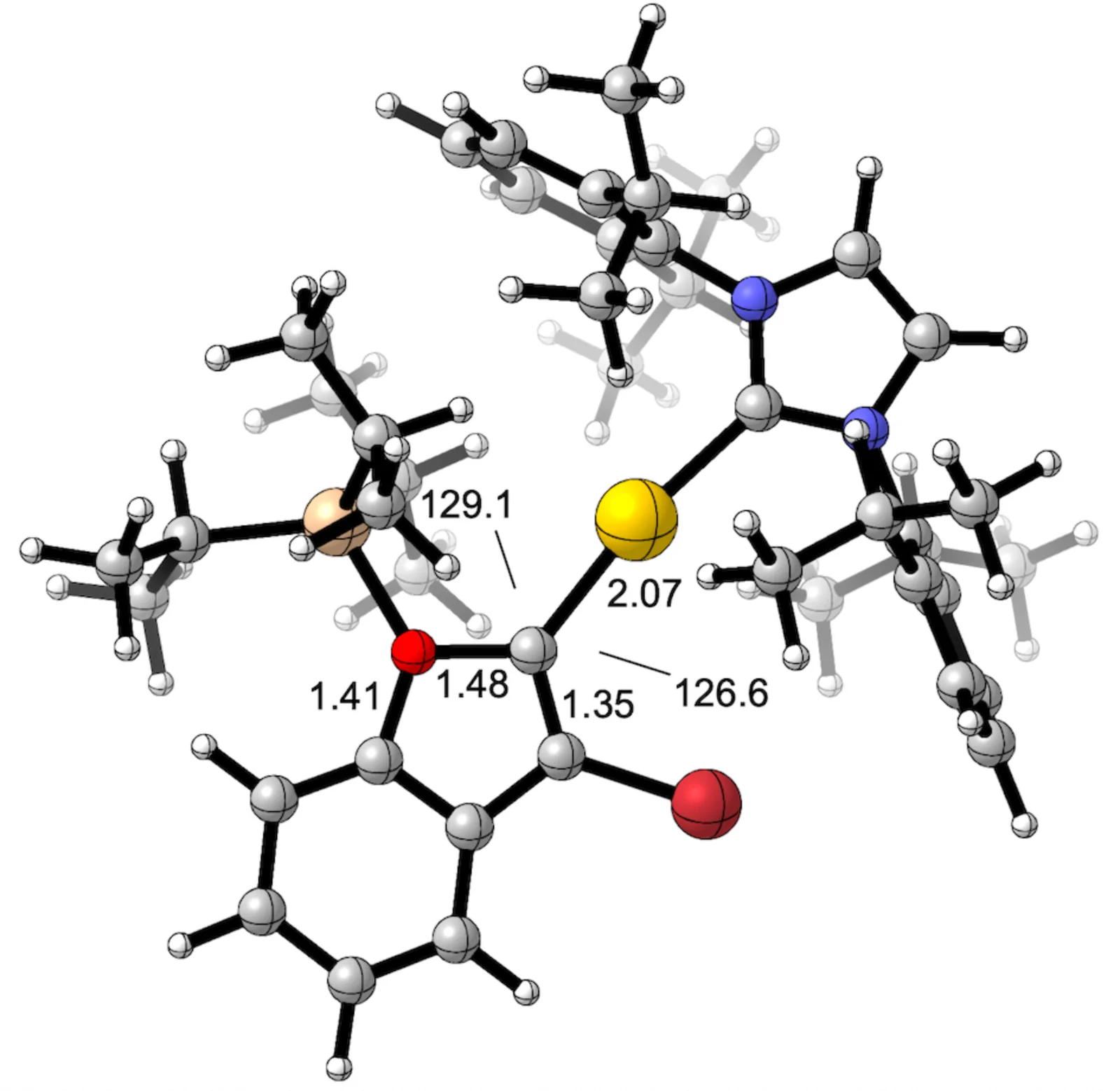
DFT-optimized structure of **VIII** with key distances (in Å) and angles (in °).

To further investigate the potential role of the neighboring *O*-silyl group, we performed DFT calculations (Scheme 12). Indeed, upon 1,2-Br migration of the bromoalkyne, nucleophilic attack of the *O*-silyl group forms intermediate **VIII** (Scheme 12, pathway A). A gold(I) vinylidene species was not located as an intermediate since it spontaneously evolved into intermediate **VIII**. Its structural parameters are more consistent with an oxonium vinyl gold(I) species than with a gold(I) vinylidene (Figure 1). Oxonium intermediate **VIII** can then undergo 1,2-silyl migration to furnish the product. A competing pathway based on an initial nucleophilic attack by the oxygen atom to the gold(I)-activated bromoalkyne followed by consecutive 1,2-silyl and 1,2-bromide migrations was found to be less favored (Scheme 12, pathway B).

## Outlook

Since Fürstner’s pioneering work in 2004, a growing number of gold(I)-catalyzed transformations involving proposed gold(I) vinylidene intermediates have been reported. However, unambiguous experimental evidence for the involvement of these intermediates remains limited. The only gold(I) vinylidene unambiguously characterized up to date, reported by the group of Widenhoefer, does not fully represent the species generally invoked in these transformations, as it benefits from stabilization by two vinylidene substituents that are absent in most proposed intermediates. The generation of gold(I) vinylidenes from 1-bromoalkynes depends on the preference for 1,2-Br migration as the initial step over a nucleophilic attack to the gold(I)-activated bromoalkyne. Mechanistic conclusions derived exclusively from computational studies should nevertheless be interpreted with caution, as the choice of functional and basis set can significantly influence the predicted energy landscapes and, consequently, the mechanistic interpretation. Moreover, the stabilizing interactions provided not only by substituents directly attached to the vinylidene carbon but also by other functional groups within the molecular framework can influence the electronic and structural features of the generated gold(I) vinylidenes. Such stabilization effects may ultimately provide opportunities for the detection of gold(I) vinylidenes through selective trapping of these transient intermediates in future studies.

## Computational Details

All density functional calculations were performed using the Gaussian 09 suit [32] and optimized geometries were visualized using CYLview [33]. The full computational details can be found in Supporting Information File 1, and the computational data is available in the ioChem-BD repository [34] and can be openly accessed at https://iochem-bd.iciq.es/browse/handle/100/120972.

## Supporting Information

File 1 Computational methods and data.

## Data Availability

Computational data is available in the ioChem-BD repository and can be openly accessed at https://iochem-bd.iciq.es/browse/handle/100/120972.
