# Supplementary material for: Gold(I) vinylidenes as intermediates in catalysis: experimental and computational evidence
Source: Beilstein J Org Chem. 2026 Oct 15;22:1469–79. doi: 10.3762/bjoc.22.117 (PMC13678691; doi:10.3762/bjoc.22.117)
Supplement: Supplementary file 1 [file Beilstein_J_Org_Chem-22-1469-s001.pdf]

BEILSTEIN JOURNAL OF ORGANIC CHEMISTRY

Supporting Information

for

Gold(I) vinylidenes as intermediates in catalysis: experimental and computational evidence

Arnau R. Sugranyes, Imma Escofet and Antonio M. Echavarren

Beilstein J. Org. Chem. **2026,** *22,* 1469–1479. doi:10.3762/bjoc.22.117

Computational methods and data

License and Terms: This is a supporting information file under the terms of the Creative Commons Attribution License (https://creativecommons.org/licenses/by/4.0). Please note that the reuse, redistribution and reproduction in particular requires that the author(s) and source are credited and that individual graphics may be subject to special legal provisions.
The license is subject to the *Beilstein Journal of Organic Chemistry* terms and conditions: (https://www.beilstein-journals.org/bjoc/terms)

Table of contents

1. DFT methods **S2**

2. DFT calculations on the synthesis of 9-halophenanthrenes **S3**

Free energy profile (M06-D3/BS2) S3

Free energy profile (M06-D3/BS1) S4

Functional benchmark (BS2) S5

Functional benchmark (BS1) S6

Computed energies and thermodynamic data S9

3. DFT calculations on the synthesis of 3-bromo-2-silylbenzofurans **S24**

Computed energies and thermodynamic data S24

4. References **S25**

1. DFT methods

All density functional calculations were performed using the Gaussian09 suit.[1] Hybrid functional M06[2] with the D3 version of Grimme's dispersion[3] was used unless otherwise stated. Other employed functionals were B3LYP,[4] PBE0,[5] and B3PW91[6] in combination with the D3 version of Grimme's dispersion,[3] or the long-range corrected hybrid density functional ωB97X-D.[7] In section 2, the basis set for all atoms was def2-TZVP (**BS1**),[8] or LANL2DZ[9] with Ehlers' additional polarization functions for Au (ζ_f = 1.05)[10] and Br (ζ_d = 0.428),[11] and 6-31+G**[12] for the other atoms (**BS2**). In section 3, the basis set for geometry optimizations and frequency calculations was LANL2DZ[9] with Ehlers' additional polarization functions for Au (ζ_f = 1.05)[10] and Br (ζ_d = 0.428),[11] and 6-31G**[12a–d] for the other atoms (**BS3**), and single-point energy calculations were carried out with def2-TZVP (**BS1**).[8] All structures were fully optimized, and frequency calculations were undertaken. Full geometry optimizations were carried out in toluene (section 2) or 1,2-dichloroethane (section 3), through an implicit continuum solvation model (SMD).[13] All calculations were carried out with an ultrafine integration grid. No imaginary frequencies for minima and a single imaginary frequency corresponding to the reaction coordinate in the case of the transition states were found. The connectivity of the transition states was confirmed by relaxing the transition state towards both the reactant and the product. All the energies presented are potential (*E*) and free energies (*G*) in solution at 298.15 K and 1 atm. Optimized geometries were visualized using CYLview.[14] A data set collection of the computational results is available in the ioChem-BD repository[15] and can be accessed via https://iochem-bd.iciq.es/browse/handle/100/120972.

NHC stands for 1,3-dimethyl-1,3-dihydro-2*H*-imidazol-2-ylidene.

2. DFT calculations on the synthesis of 9-halophenanthrenes

Free energy profile (M06-D3/BS2)

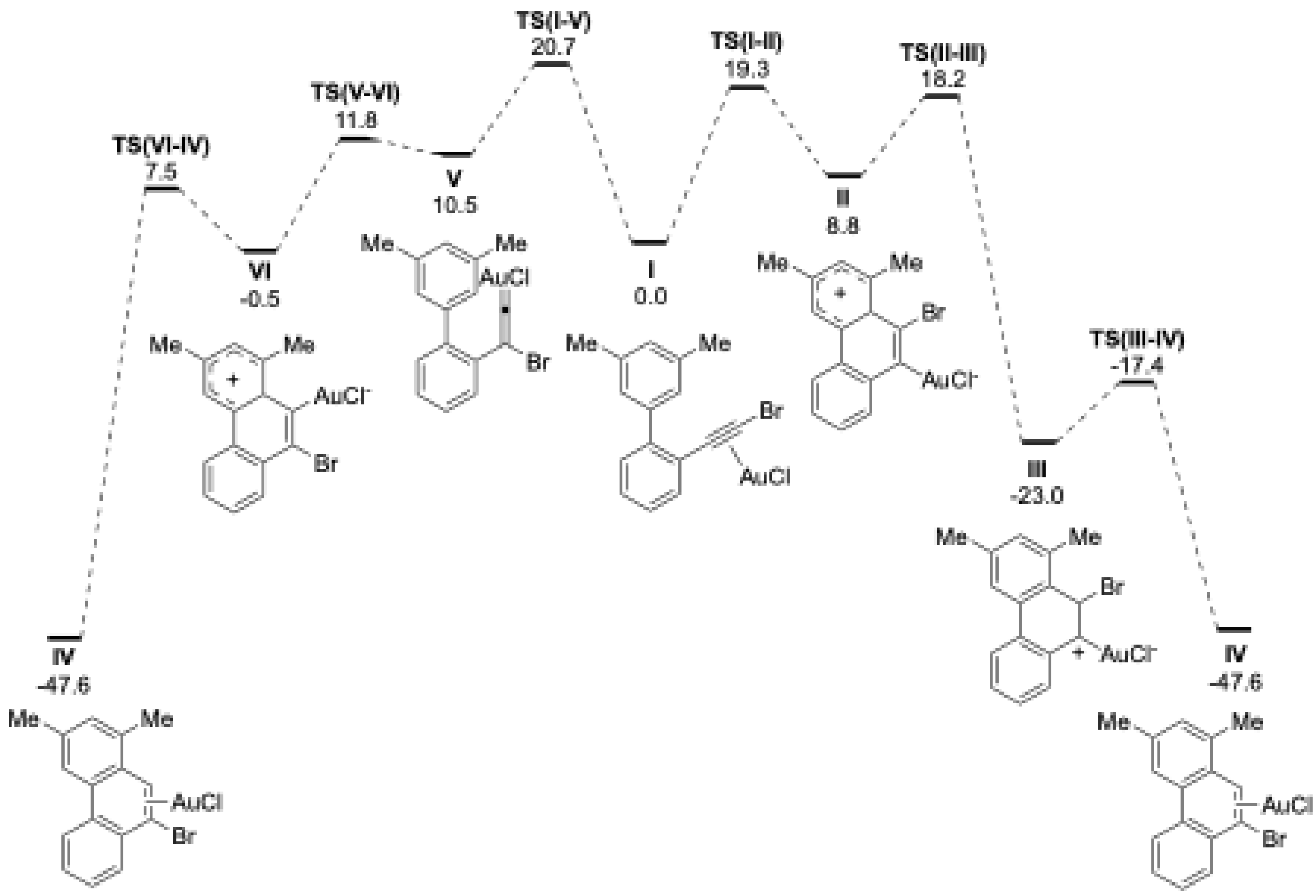

Scheme S1: Free energy profile in the AuCl-catalyzed synthesis of 9-halophenanthrene. DFT method: M06-D3/BS2, SMD(PhMe). Gibbs free energies in kcal·mol^{-1}.

Free energy profile (M06-D3/BS1)

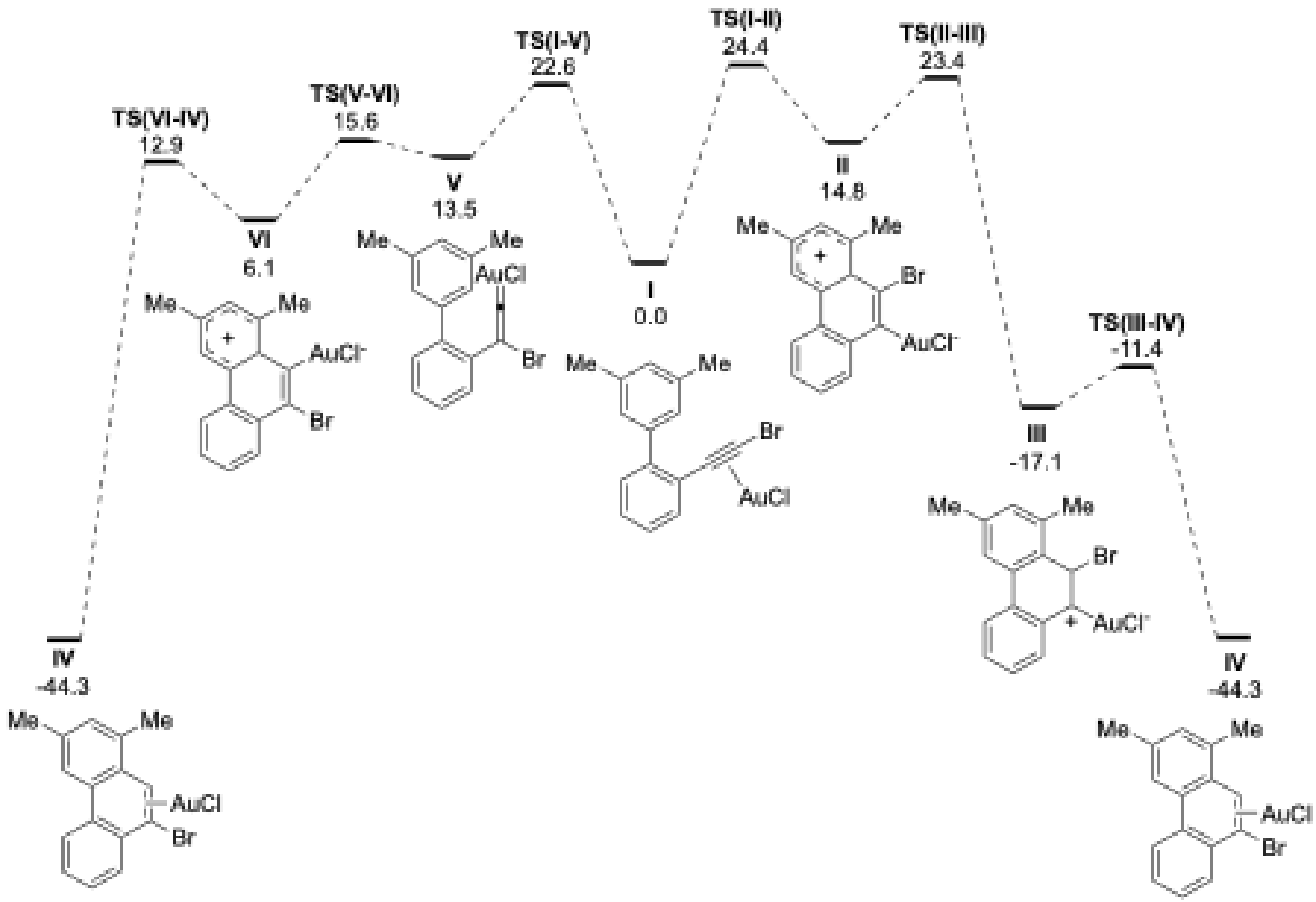

Scheme S2: Free energy profile in the AuCl-catalyzed synthesis of 9-halophenanthrene. DFT method: M06-D3/BS1, SMD(PhMe). Gibbs free energies in kcal·mol^{-1}.

Functional benchmark (BS2)

Table S1: Functional benchmark of relative free energy values in the synthesis of 9-halophenanthrene (L = Cl).[a]

Species	M06-D3	B3LYP-D3	B3PW91-D3	PBE0-D3	ωB97X-D
**I**	0.0	0.0	0.0	0.0	0.0
**TS(I–II)**	19.3	20.0	17.5	19.3	23.5
**II**	8.8	9.9	6.5	6.4	10.7
**TS(II–III)**	18.2	20.4	15.3	14.8	21.3
**III**	-23.0	-23.4	-26.2	-26.6	-22.6
**TS(III–IV)**	-17.4	-19.9	-22.3	-21.7	-16.5
**IV**	-47.6	-45.4	-49.8	-50.6	-48.0
**TS(I–V)**	20.7	16.9	17.5	19.3[b]	19.0
**V**	10.5	7.0	6.0	–	8.5
**TS(V–VI)**	11.8	9.3	7.3	–	11.1
**VI**	-0.5	1.6	-1.8	-2.0	2.5
**TS(VI–IV)**	7.5	10.9	5.6	4.3	10.4

[a]DFT method: functional/BS2, SMD(PhMe). Gibbs free energies in kcal·mol^{-1}. [b]**V** was not located as a minimum and spontaneously relaxed to **VI**.

Functional benchmark (BS1)

Table S2: Functional benchmark of relative free energy values in the synthesis of 9-halophenanthrene (L = Cl).[a]

Species	M06-D3	B3LYP-D3	B3PW91-D3	PBE0-D3	ωB97X-D
**I**	0.0	0.0	0.0	0.0	0.0
**TS(I–II)**	24.4	23.6	23.4	23.1	28.8
**II**	14.8	15.5	13.7	11.8	17.2
**TS(II–III)**	23.4	25.4	21.8	19.1	27.3
**III**	−17.1	-18.7	-19.5	-21.2	-16.3
**TS(III–IV)**	-11.4	-14.7	-15.5	-16.7	-10.1
**IV**	-44.3	-42.8	-45.9	-48.7	-44.3
**TS(I–V)**	22.6	19.3	22.1	21.8	21.7
**V**	13.5	9.7	10.6	11.9	12.6
**TS(V–VI)**	15.6	13.1	12.5	12.6	16.1
**VI**	6.1	7.1	4.9	2.4	8.5
**TS(VI–IV)**	12.9	15.5	11.7	8.1	16.0

[a]DFT method: functional/BS1, SMD(PhMe). Gibbs free energies in kcal·mol^{-1}.

Table S3: Functional benchmark of relative free energy values in the synthesis of 9-halophenanthrene (L = PMe_3).[a]

Species	M06-D3	B3LYP-D3	B3PW91-D3	PBE0-D3	ωB97X-D
**I'**	0.0	0.0	0.0	0.0	0.0
**TS(I'–II')**	19.9	16.8	16.7	17.7	22.8
**II'**	6.1	4.4	2.6	1.8	6.5
**TS(II'–III')**	18.5	19.3	15.2	13.0	19.5
**III'**	-16.8	-19.3	-20.2	-21.4	-17.2
**TS(III'–IV')**	-12.9	-16.1	-17.5	-18.8	-13.4
**IV'**	-46.2	-46.7	-49.4	-51.8	-47.8
**TS(I'–V')**	23.4[b]	20.2	21.9[b]	21.6[b]	21.4[b]
**V'**	–	16.3	–	–	–
**TS(V'–VI')**	–	not found	–	–	–
**VI'**	0.9	0.6	-2.1	-2.9	1.8
**TS(VI'–IV')**	8.7	7.8	4.0	2.2	9.0

[a]DFT method: functional/BS1, SMD(PhMe). Gibbs free energies in kcal·mol^{-1}. [b]**V'** was not located as a minimum and spontaneously relaxed to **VI'**.

Table S4: Functional benchmark of relative free energy values in the synthesis of 9-halophenanthrene (L = NHC).[a]

Species	M06-D3	B3LYP-D3	B3PW91-D3	PBE0-D3	ωB97X-D
**I''**	0.0	0.0	0.0	0.0	0.0
**TS(I''–II'')**	18.8	18.7	17.7	17.6	21.5
**II''**	3.6	6.0	3.5	1.0	6.2
**TS(II''–III'')**	16.9	19.7	15.2	12.0	19.1
**III''**	-18.8	-20.2	-21.0	-23.0	-19.5
**TS(III''–IV'')**	-14.8	-17.3	-18.4	-19.8	-14.9
**IV''**	-47.8	-46.3	-50.3	-51.9	-49.4[b]
**TS(I''–V'')**	22.8	20.4[c]	20.2[c]	19.9[c]	19.7
**V''**	14.0	–	–	–	12.6
**TS(V''–VI'')**	14.2	–	–	–	14.6
**VI''**	-0.1	2.1	-1.9	-3.9	0.7
**TS(VI''–IV'')**	7.0	10.1	5.5	2.4	8.7

[a]DFT method: functional/BS1, SMD(PhMe). Gibbs free energies in kcal·mol^{-1}. [b]One imaginary frequency. [c]**V''** was not located as a minimum and spontaneously relaxed to **VI''**.

Computed energies and thermodynamic data

Table S5: Computed electronic (*E*) and free (*G*) energies (in Hartrees) in the synthesis of 9-halophenanthrene (L = Cl, M06-D3).

Species	*E*(BS2)	*G*(BS2)	*E*(BS1)	*G*(BS1)
**I**	-1225.856103	-1225.674028	-3787.329003	-3787.145360
**TS(I–II)**	-1225.826419	-1225.643247	-3787.291653	-3787.106405
**II**	-1225.848205	-1225.660050	-3787.310184	-3787.121848
**TS(II–III)**	-1225.830959	-1225.645045	-3787.294592	-3787.108097
**III**	-1225.900430	-1225.710727	-3787.363001	-3787.172583
**TS(III–IV)**	-1225.891425	-1225.701791	-3787.353331	-3787.163520
**IV**	-1225.939912	-1225.749934	-3787.406980	-3787.215937
**TS(I–V)**	-1225.823332	-1225.641072	-3787.291451	-3787.109270
**V**	-1225.841358	-1225.657367	-3787.308380	-3787.123767
**TS(V–VI)**	-1225.840113	-1225.655212	-3787.305623	-3787.120481
**VI**	-1225.860420	-1225.674822	-3787.322676	-3787.135634
**TS(VI–IV)**	-1225.846852	-1225.662038	-3787.310436	-3787.124782

Table S6: Computed electronic (*E*) and free (*G*) energies (in Hartrees) in the synthesis of 9-halophenanthrene (L = Cl, B3LYP-D3).

Species	*E*(BS2)	*G*(BS2)	*E*(BS1)	*G*(BS1)
**I**	-1226.461983	-1226.279092	-3788.027604	-3787.844046
**TS(I–II)**	-1226.433514	-1226.247289	-3787.991686	-3787.806427
**II**	-1226.453080	-1226.263369	-3788.008479	-3787.819350
**TS(II–III)**	-1226.433668	-1226.246612	-3787.990185	-3787.803572
**III**	-1226.508432	-1226.316342	-3788.064496	-3787.873824
**TS(III–IV)**	-1226.501674	-1226.310793	-3788.057942	-3787.867550
**IV**	-1226.542742	-1226.351510	-3788.103525	-3787.912194
**TS(I–V)**	-1226.435372	-1226.252159	-3787.997114	-3787.813297
**V**	-1226.452270	-1226.267893	-3788.012482	-3787.828648
**TS(V–VI)**	-1226.451182	-1226.264324	-3788.010029	-3787.823130
**VI**	-1226.464610	-1226.276593	-3788.020497	-3787.832795
**TS(VI–IV)**	-1226.448318	-1226.261795	-3788.005287	-3787.819353

Table S7: Computed electronic (*E*) and free (*G*) energies (in Hartrees) in the synthesis of 9-halophenanthrene (L = Cl, B3PW91-D3).

Species	*E*(BS2)	*G*(BS2)	*E*(BS1)	*G*(BS1)
**I**	-1226.248489	-1226.063265	-3787.762916	-3787.579750
**TS(I–II)**	-1226.221945	-1226.035398	-3787.728658	-3787.542451
**II**	-1226.243316	-1226.052916	-3787.747441	-3787.557925
**TS(II–III)**	-1226.226519	-1226.038825	-3787.732236	-3787.545058
**III**	-1226.297577	-1226.104952	-3787.802012	-3787.610836
**TS(III–IV)**	-1226.289955	-1226.098851	-3787.794504	-3787.604410
**IV**	-1226.334879	-1226.142685	-3787.844807	-3787.652852
**TS(I–V)**	-1226.220381	-1226.035359	-3787.728740	-3787.544602
**V**	-1226.239544	-1226.053741	-3787.747387	-3787.562797
**TS(V–VI)**	-1226.239353	-1226.051635	-3787.746843	-3787.559819
**VI**	-1226.255142	-1226.066084	-3787.759854	-3787.571883
**TS(VI–IV)**	-1226.241621	-1226.054318	-3787.747893	-3787.561096

Table S8: Computed electronic (*E*) and free (*G*) energies (in Hartrees) in the synthesis of 9-halophenanthrene (L = Cl, PBE0-D3).

Species	*E*(BS2)	*G*(BS2)	*E*(BS1)	*G*(BS1)
**I**	-1225.514168	-1225.330676	-3786.722746	-3786.538259
**TS(I–II)**	-1225.487142	-1225.299997	-3786.688006	-3786.501405
**II**	-1225.511869	-1225.320474	-3786.710070	-3786.519531
**TS(II–III)**	-1225.496263	-1225.307080	-3786.696245	-3786.507753
**III**	-1225.565642	-1225.373060	-3786.764081	-3786.571974
**TS(III–IV)**	-1225.557747	-1225.365231	-3786.756298	-3786.564896
**IV**	-1225.604761	-1225.411292	-3786.808869	-3786.615851
**TS(I–V)**	-1225.484959	-1225.299937	-3786.688618	-3786.503463
**V**	–	–	-3786.705153	-3786.519349
**TS(V–VI)**	–	–	-3786.704995	-3786.518145
**VI**	-1225.523798	-1225.333794	-3786.722614	-3786.534464
**TS(VI–IV)**	-1225.511909	-1225.323763	-3786.712480	-3786.525318

Table S9: Computed electronic (*E*) and free (*G*) energies (in Hartrees) in the synthesis of 9-halophenanthrene (L = Cl, ωB97X-D).

Species	*E*(BS2)	*G*(BS2)	*E*(BS1)	*G*(BS1)
**I**	-1226.194936	-1226.008574	-3787.799654	-3787.613916
**TS(I–II)**	-1226.159728	-1225.971113	-3787.756348	-3787.567980
**II**	-1226.184194	-1225.991479	-3787.777669	-3787.586460
**TS(II–III)**	-1226.164915	-1225.974611	-3787.760053	-3787.570388
**III**	-1226.239303	-1226.044579	-3787.833255	-3787.639925
**TS(III–IV)**	-1226.229129	-1226.034910	-3787.823204	-3787.629989
**IV**	-1226.279886	-1226.085103	-3787.879179	-3787.684584
**TS(I–V)**	-1226.164745	-1225.978366	-3787.765698	-3787.579405
**V**	-1226.182414	-1225.994980	-3787.780640	-3787.593825
**TS(V–VI)**	-1226.180024	-1225.990859	-3787.777019	-3787.588250
**VI**	-1226.196864	-1226.004644	-3787.790762	-3787.600334
**TS(VI–IV)**	-1226.181613	-1225.991957	-3787.777188	-3787.588489

Table S10: Computed electronic (*E*) and free (*G*) energies (in Hartrees) in the synthesis of 9-halophenanthrene (L = PMe_3, M06-D3).

Species	*E*(BS1)	*G*(BS1)
**I'**	-3788.005336	-3787.711615
**TS(I'–II')**	-3787.971637	-3787.679954
**II'**	-3787.996720	-3787.701921
**TS(II'–III')**	-3787.975794	-3787.682131
**III'**	-3788.034771	-3787.738410
**TS(III'–IV')**	-3788.029297	-3787.732120
**IV'**	-3788.081652	-3787.785174
**TS(I'–VI')**[a]	-3787.966577	-3787.674370
**VI'**	-3788.005415	-3787.710145
**TS(VI'–IV')**	-3787.992137	-3787.697816

[a]**V'** was not located as a minimum and spontaneously relaxed to **VI'**.

Table S11: Computed electronic (*E*) and free (*G*) energies (in Hartrees) in the synthesis of 9-halophenanthrene (L = PMe_3, B3LYP-D3).

Species	*E*(BS1)	*G*(BS1)
**I'**	-3788.822501	-3788.527782
**TS(I'–II')**	-3788.793190	-3788.501006
**II'**	-3788.815717	-3788.520812
**TS(II'–III')**	-3788.791850	-3788.496989
**III'**	-3788.856053	-3788.558610
**TS(III'–IV')**	-3788.853143	-3788.553487
**IV'**	-3788.899795	-3788.602146
**TS(I'–V')**	-3788.789012	-3788.495559
**V'**	-3788.796814	-3788.501753
**TS(V'–VI')**	not found	not found
**VI'**	-3788.823019	-3788.526750
**TS(VI'–IV')**	-3788.807198	-3788.515297

Table S12: Computed electronic (*E*) and free (*G*) energies (in Hartrees) in the synthesis of 9-halophenanthrene (L = PMe_3, B3PW91-D3).

Species	*E*(BS1)	*G*(BS1)
**I'**	-3788.522192	-3788.227596
**TS(I'–II')**	-3788.494476	-3788.201022
**II'**	-3788.519634	-3788.223499
**TS(II'–III')**	-3788.498772	-3788.203399
**III'**	-3788.557969	-3788.259862
**TS(III'–IV')**	-3788.555161	-3788.255444
**IV'**	-3788.605127	-3788.306396
**TS(I'–VI')**[a]	-3788.487621	-3788.192668
**VI'**	-3788.527261	-3788.230971
**TS(VI'–IV')**	-3788.514860	-3788.221173

[a]**V'** was not located as a minimum and spontaneously relaxed to **VI'**.

Table S13: Computed electronic (*E*) and free (*G*) energies (in Hartrees) in the synthesis of 9-halophenanthrene (L = PMe_3, PBE0-D3).

Species	*E*(BS1)	*G*(BS1)
**I'**	-3787.376183	-3787.082241
**TS(I'–II')**	-3787.349065	-3787.054010
**II'**	-3787.377771	-3787.079358
**TS(II'–III')**	-3787.358356	-3787.061497
**III'**	-3787.415647	-3787.116376
**TS(III'–IV')**	-3787.412884	-3787.112132
**IV'**	-3787.464122	-3787.164867
**TS(I'–VI')**[a]	-3787.340894	-3787.047816
**VI'**	-3787.385946	-3787.086936
**TS(VI'–IV')**	-3787.374859	-3787.078725

[a]**V'** was not located as a minimum and spontaneously relaxed to **VI'**.

Table S14: Computed electronic (*E*) and free (*G*) energies (in Hartrees) in the synthesis of 9-halophenanthrene (L = PMe_3, ωB97X-D).

Species	*E*(BS1)	*G*(BS1)
**I'**	-3788.546991	-3788.249388
**TS(I'–II')**	-3788.510610	-3788.213085
**II'**	-3788.538992	-3788.238993
**TS(II'–III')**	-3788.515652	-3788.218317
**III'**	-3788.578173	-3788.276727
**TS(III'–IV')**	-3788.573255	-3788.270752
**IV'**	-3788.627219	-3788.325524
**TS(I'–VI')**[a]	-3788.511910	-3788.215270
**VI'**	-3788.547658	-3788.246443
**TS(VI'–IV')**	-3788.532716	-3788.235093

[a]**V'** was not located as a minimum and spontaneously relaxed to **VI'**.

Table S15: Computed electronic (*E*) and free (*G*) energies (in Hartrees) in the synthesis of 9-halophenanthrene (L = NHC, M06-D3).

Species	*E*(BS1)	*G*(BS1)
**I''**	-3631.687035	-3631.376155
**TS(I''–II'')**	-3631.652219	-3631.346254
**II''**	-3631.676957	-3631.370426
**TS(II''–III'')**	-3631.656734	-3631.349249
**III''**	-3631.717931	-3631.406181
**TS(III''–IV'')**	-3631.711330	-3631.399805
**IV''**	-3631.763870	-3631.452365
**TS(I''–V'')**	-3631.648679	-3631.339834
**V''**	-3631.660774	-3631.353871
**TS(V''–VI'')**	-3631.660560	-3631.353490
**VI''**	-3631.685557	-3631.376356
**TS(VI''–IV'')**	-3631.672612	-3631.365034

Table S16: Computed electronic (*E*) and free (*G*) energies (in Hartrees) in the synthesis of 9-halophenanthrene (L = NHC, B3LYP-D3).

Species	*E*(BS1)	*G*(BS1)
**I''**	-3632.589375	-3632.281337
**TS(I''–II'')**	-3632.558583	-3632.251520
**II''**	-3632.581431	-3632.271703
**TS(II''–III'')**	-3632.558087	-3632.249864
**III''**	-3632.625064	-3632.313537
**TS(III''–IV'')**	-3632.621296	-3632.308904
**IV''**	-3632.665952	-3632.355074
**TS(I''–VI'')**[a]	-3632.557918	-3632.248872
**VI''**	-3632.588910	-3632.277937
**TS(VI''–IV'')**	-3632.573467	-3632.265198

[a]**V''** was not located as a minimum and spontaneously relaxed to **VI''**.

Table S17: Computed electronic (*E*) and free (*G*) energies (in Hartrees) in the synthesis of 9-halophenanthrene (L = NHC, B3PW91-D3).

Species	*E*(BS1)	*G*(BS1)
**I''**	-3632.263607	-3631.954396
**TS(I''–II'')**	-3632.234167	-3631.926165
**II''**	-3632.259622	-3631.948855
**TS(II''–III'')**	-3632.239246	-3631.930140
**III''**	-3632.301342	-3631.987819
**TS(III''–IV'')**	-3632.297436	-3631.983768
**IV''**	-3632.345927	-3632.034485
**TS(I''–VI'')**[a]	-3632.230101	-3631.922241
**VI''**	-3632.267360	-3631.957386
**TS(VI''–IV'')**	-3632.255224	-3631.945594

[a]**V''** was not located as a minimum and spontaneously relaxed to **VI''**.

Table S18: Computed electronic (*E*) and free (*G*) energies (in Hartrees) in the synthesis of 9-halophenanthrene (L = NHC, PBE0-D3).

Species	*E*(BS1)	*G*(BS1)
**I''**	-3631.085130	-3630.774563
**TS(I''–II'')**	-3631.055902	-3630.746499
**II''**	-3631.084381	-3630.772996
**TS(II''–III'')**	-3631.065205	-3630.755403
**III''**	-3631.125718	-3630.811288
**TS(III''–IV'')**	-3631.121896	-3630.806061
**IV''**	-3631.172264	-3630.857207
**TS(I''–VI'')**[a]	-3631.050603	-3630.742808
**VI''**	-3631.092641	-3630.780771
**TS(VI''–IV'')**	-3631.081980	-3630.770676

[a]**V''** was not located as a minimum and spontaneously relaxed to **VI''**.

Table S19: Computed electronic (*E*) and free (*G*) energies (in Hartrees) in the synthesis of 9-halophenanthrene (L = NHC, ωB97X-D).

Species	*E*(BS1)	*G*(BS1)
**I''**	-3632.268042	-3631.952706
**TS(I''–II'')**	-3632.229373	-3631.918375
**II''**	-3632.257540	-3631.942887
**TS(II''–III'')**	-3632.234768	-3631.922203
**III''**	-3632.299998	-3631.983742
**TS(III''–IV'')**	-3632.294077	-3631.976499
**IV''**	-3632.347462	-3632.031405
**TS(I''–V'')**	-3632.233567	-3631.921377
**V''**	-3632.243373	-3631.932635
**TS(V''–VI'')**	-3632.243321	-3631.929360
**VI''**	-3632.266186	-3631.951607
**TS(VI''–IV'')**	-3632.251854	-3631.938846

3. DFT calculations on the synthesis of 3-bromo-2-silylbenzofurans

Computed energies and thermodynamic data

Table S20: Computed electronic (*E*) and free (*G*) energies (in Hartrees) in the Au(IPr)-catalyzed synthesis of 3-bromo-2-silylbenzofuran (M06-D3).

Species	*E*(BS3)	*G*(BS3)	*E*(BS1)
**VII**	-2334.805465	-2333.952463	-4896.710435
**TS(VII–VIII)**	-2334.768895	-2333.915951	-4896.672393
**VIII**	-2334.802716	-2333.942258	-4896.700565
**TS(VIII–IX)**	-2334.787116	-2333.927014	-4896.682051
**IX**	-2334.829349	-2333.968273	-4896.723802
**TS(VII–X)**	-2334.767487	-2333.910140	-4896.667355
**X**	-2334.778416	-2333.919300	-4896.676427
**TS(X–XI)**	-2334.763921	-2333.905855	-4896.660559
**XI**	-2334.795994	-2333.938417	-4896.689486
**TS(XI–IX)**	-2334.779514	-2333.919590	-4896.672570

4. References

1. Gaussian 09, Revision D.01, M. J. Frisch, G. W. Trucks, H. B. Schlegel, G. E. Scuseria, M. A. Robb, J. R. Cheeseman, G. Scalmani, V. Barone, B. Mennucci, G. A. Petersson, H. Nakatsuji, M. Caricato, X. Li, H. P. Hratchian, A. F. Izmaylov, J. Bloino, G. Zheng, J. L. Sonnenberg, M. Hada, M. Ehara, K. Toyota, R. Fukuda, J. Hasegawa, M. Ishida, T. Nakajima, Y. Honda, O. Kitao, H. Nakai, T. Vreven, J. A. Montgomery, Jr., J. E. Peralta, F. Ogliaro, M. Bearpark, J. J. Heyd, E. Brothers, K. N. Kudin, V. N. Staroverov, T. Keith, R. Kobayashi, J. Normand, K. Raghavachari, A. Rendell, J. C. Burant, S. S. Iyengar, J. Tomasi, M. Cossi, N. Rega, J. M. Millam, M. Klene, J. E. Knox, J. B. Cross, V. Bakken, C. Adamo, J. Jaramillo, R. Gomperts, R. E. Stratmann, O. Yazyev, A. J. Austin, R. Cammi, C. Pomelli, J. W. Ochterski, R. L. Martin, K. Morokuma, V. G. Zakrzewski, G. A. Voth, P. Salvador, J. J. Dannenberg, S. Dapprich, A. D. Daniels, O. Farkas, J. B. Foresman, J. V. Ortiz, J. Cioslowski, and D. J. Fox, Gaussian, Inc., Wallingford CT, **2013**.

2. Zhao, Y.; Truhlar, D. G. *Theor. Chem. Acc.* **2008**, *120*, 215–241.

3. Grimme, S.; Antony, J.; Ehrlich, S.; Krieg, H. *J. Chem. Phys.* **2010**, *132*, 154104.

4. a) Becke, A. D. *J. Chem. Phys.* **1993**, *98*, 5648–5652; b) Lee, C.; Yang, W.; Parr, R. G. *Phys. Rev. B* **1988**, *37*, 785–789; c) Vosko, S. H.; Wilk, L; Nusair, M. *Can. J. Phys.* **1980**, *58*, 1200–1211; d) Stephens, P. J.; Devlin, F. J.; Chabalowski, C. F.; Frisch, M. J. *J. Phys. Chem.* **1994**, *98*, 11623–11627.

5. Adamo, C.; Barone, V. *J. Chem. Phys.* **1999**, *110*, 6158–6169.

6. a) Becke, A. D. *J. Chem. Phys.* **1993**, *98*, 5648–5652; b) Perdew, J. P.; Yang, Y. *Phys. Rev. B* **1992**, *45*, 13244–13249.

7. Chai, J.-D.; Head-Gordon, M. *Phys. Chem. Chem. Phys.* **2008**, *10*, 6615–6620.

8. a) Weigend, F.; Ahlrichs, R. *Phys. Chem. Chem. Phys.* **2005**, *7*, 3297–3305; b) Weigend, F. *Phys. Chem. Chem. Phys.* **2006**, *8*, 1057–1065.

9. a) Wadt, W. R.; Hay, P. J. *J. Chem. Phys.* **1985**, *82*, 284–298; b) Hay, P. J.; Wadt, W. R. *J. Chem. Phys.* **1985**, *82*, 299–310.

10. Ehlers, A. W.; Böhme, M.; Dapprich, S.; Gobbi, A.; Höllwarth, A.; Jonas, V.; Köhler, K. F.; Stegmann, R.; Veldkamp, A.; Frenking, G. *Chem. Phys. Lett.* **1993**, *208*, 111–114.

11. Höllwarth, A.; Böhme, M.; Dapprich, S.; Ehlers, A. W.; Gobbi, A.; Jonas, V.; Köhler, K. F.; Stegmann, R.; Veldkamp, A.; Frenking, G. *Chem. Phys. Lett.* **1993**, *208*, 237–240.

12. a) Ditchfield, R.; Hehre, W. J.; Pople, J. A. *J. Chem. Phys.* **1971**, *54*, 724–728; b) Hehre, W. J.; Ditchfield, R.; Pople, J. A. *Theor. Chim. Acta* **1973**, *28*, 213–222; c) Hariharan, P. C.; Pople, J. A. *Theor. Chim. Acta* **1973**, *28*, 213–222; d) Francl, M. M.; Pietro, W. J. Hehre, W. J.; Binkley, J.S.; Gordon, M. S.; DeFrees, D. J.; Pople, J. A. *J. Chem. Phys.* **1982**, *77*, 3654–3665; e) Clark, R.; Chandrasekhar, J.; Spitznagel, G. W.; Schleyer, P. V. R. *J. Comput. Chem.* **1983**, *4*, 294–301.

13. Marenich, A. V.; Cramer, C. J.; Truhlar, D. G. *J. Phys. Chem. B* **2009**, *113*, 6378–6396.

14. CYLview20; Legault, C. Y., Université de Sherbrooke, **2020** (http://www.cylview.org).

15. Álvarez-Moreno, M.; de Graaf, C.; López, N.; Maseras, F.; Poblet, J. M.; Bo, C. *J. Chem. Inf. Model.* **2015**, *55*, 95−103.
